# Supplementary material for: Adenosine A2A Receptor Antagonists Affects NMDA Glutamate Receptor Function. Potential to Address Neurodegeneration in Alzheimer’s Disease
Source: Cells. 2020 Apr 26;9(5):1075. doi: 10.3390/cells9051075 (PMC7290564; doi:10.3390/cells9051075)
Supplement: Supplementary file 1 [file cells-09-01075-s001.pdf]

cells

MDPI

Original Article

Supplementary Material

Adenosine A_{2A} Receptor Antagonists Affects NMDA Glutamate Receptor Function. Potential to Address Neurodegeneration in Alzheimer's Disease

Rafael Franco *, Rafael Rivas-Santisteban, Mireia Casanovas, Alejandro Lillo, Carlos A. Saura and Gemma Navarro *

* Correspondence: rfranco123@gmail.com (R.F.); g.navarro@ub.edu (G.N.)

Abstract: (1) Background. *N*-methyl D-aspartate (NMDA) ionotropic glutamate receptor (NMDAR), which is one of the main targets to combat Alzheimer's disease (AD), is expressed in both neurons and glial cells. The aim of this paper was to assess whether the adenosine A_{2A} receptor ($A_{2A}R$), which is a target in neurodegeneration, may affect NMDAR functionality. (2) Methods. Immuno-histo/cytochemical, biophysical, biochemical and signaling assays were performed in a heterologous cell expression system and in primary cultures of neurons and microglia (resting and activated) from control and the $APP_{Sw,Ind}$ transgenic mice. (3) Results. On the one hand, NMDA and A_{2A} receptors were able to physically interact forming complexes, mainly in microglia. Furthermore, the amount of complexes was markedly enhanced in activated microglia. On the other hand, the interaction resulted in a novel functional entity that displayed a cross-antagonism, that could be useful to prevent the exacerbation of NMDAR function by using $A_{2A}R$ antagonists. Interestingly, the amount of complexes was markedly higher in the hippocampal cells from the $APP_{Sw,Ind}$ than from the control mice. In neurons, the number of complexes was lesser, probably due to NMDAR not interacting with the $A_{2A}R$. However, the activation of the $A_{2A}R$ receptors resulted in higher NMDAR functionality in neurons, probably by indirect mechanisms. (4) Conclusions. $A_{2A}R$ antagonists such as istradefylline, which is already approved for Parkinson's disease (Nouriast® in Japan and Nourianz® in the US), have potential to afford neuroprotection in AD in a synergistic-like fashion. i.e., via both neurons and microglia.

Keywords: G-protein-coupled receptors; functional selectivity; microglia; neuroprotection; cognition; signaling

Figure Supplementary 1

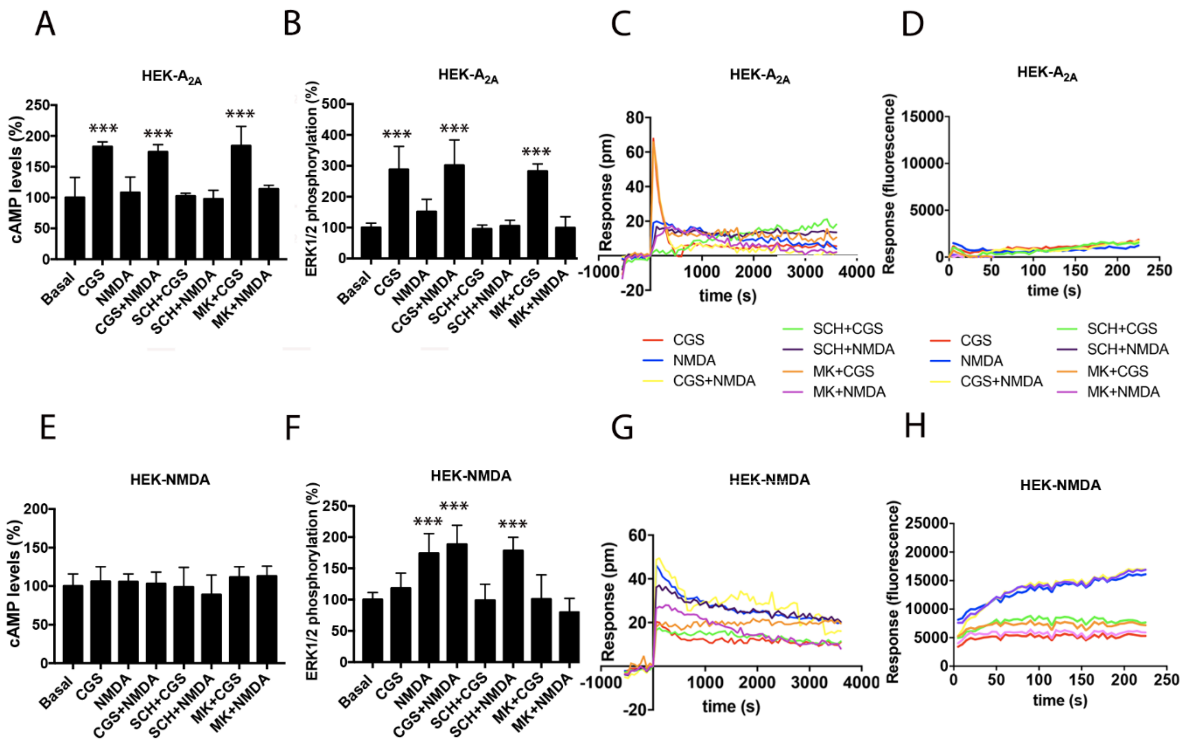

Supplementary Figure S1. Functional signaling of A_{2A} and NMDA receptors in HEK-293T cells. In (**A**) to (**H**), HEK-293T cells expressing A_{2A}R (0.5 µg of cDNA) (**A** to **C**), GluN1 (0.5 µg of cDNA) and GluN2 (0.5 µg of cDNA) (**E** to **G**), A_{2A}R (0.5 µg of cDNA) and 6GCaMP calcium sensor (0.75 µg of cDNA) (**D**) or GluN1 (0.5 µg of cDNA), GluN2 (0.5 µg of cDNA) and 6GCaMP calcium sensor (0.75 µg of cDNA) (**H**) were not stimulated, pre-stimulated with 1 µM of the A_{2A}R antagonist SCH-58261 (SCH) or 1 µM of the NMDAR antagonist, MK-801 (MK), and stimulated with 100 nM of the A_{2A}R agonist CGS-21680 (CGS) or 15 µM NMDA or both and the cAMP levels (**A**,**E**), ERK 1/2 phosphorylation signal (**B**,**F**), representative traces of Dynamic Mass Redistribution (DMR) (**C**,**G**) and representative traces of intracellular Ca^{2+} responses over time (**D**,**H**) were determined. Values are mean ± SEM of 10 to 12 different experiments. ERK 1/2 phosphorylation levels and cAMP increases are expressed as percentage over basal. One-way ANOVA followed by a Bonferroni multiple comparison post hoc test showed a significant effect over 100% (* $p < 0.05$, ** $p < 0.01$, *** $p < 0.001$).
